# 

**Published:** 1900-03-17

**Authors:** 


**DEATH.**

WOODCOCK.—On the 9th inst., at Hove, after a long illness, Emily, youngest daughter of the Rev. Canon Woodcock. For 13 years Head of the Hollond Institution, Nice. (2,556)

### NOTICE TO CORRESPONDENTS.

All MS., Letters, Books for Review, and other matters intended for the Editor should be addressed The Editor, "The Hospital," The Hospital Building, 28 & 29, Southampton Street, Strand, W.C.

The Editor cannot undertake to return rejected MS., even when accompanied by stamped directed envelope.

**Notice to Advertisers and Subscribers.**

All Advertisements, Orders for Copies of The Hospital, and Business Communications should be addressed to **THE MANAGER (Not to the Editor)**, The Hospital, The Hospital Building, 28 & 29, Southampton Street, Strand, London, W.C.

The Cost of Subscription, post free, is as follows:—Per week, 2½d.; per quarter, 2s. 8d.; per half-year, 5s. 3d.; per annum, 10s. 6d. Foreign:—Per annum, 15s. 2d., payable, in all cases, in advance.

**NOTICE.—Vol. XXVI. of "The Hospital" (April, 1899, to September, 1899), in handsome cloth binding, is now on sale at the Office of this Journal, price 7s. 6d. Cases for binding the Numbers contained in this Volume 1s. 6d. Index to Vol. XXVI., 2½d., post free.**

**The Monthly Part for March is now ready. Price 6d., by post 8d.**

### NOW READY,

A REPRINT OF

### A NURSING HANDBOOK

TOGETHER WITH S. MAW, SON, AND THOMPSON'S

### COMPLETE CATALOGUE OF NURSING REQUISITES.

The above work is now republished at a cost of 1s. per copy. Messrs. S. M., S., and T. have been compelled to make this charge on account of the extreme popularity of the work and the great expense incurred in its production.

***Post Free on receipt of 1s.***

### S. MAW, SON, and THOMPSON,

7 to 12, ALDERSGATE STREET, LONDON, E.C.

NOW READY. With 36 Illustrations, crown 8vo., cloth, 248 pp., price 6s.

### PRACTICAL TEXT-BOOK OF MIDWIFERY FOR NURSES AND STUDENTS.

By ROBERT JARDINE, M.D.Ed., M.R.C.S.Eng., F.F.P. & S. Glasgow, Physician to the Glasgow Maternity Hospital.

By the Same Author.

### CLINICAL LECTURES ON THE HÆMORRHAGES OF PREGNANCY, PARTURITION, AND THE PUERPERIUM,

AND THE TREATMENT OF ECLAMPSIA BY SALINE INFUSION, with Notes on 17 Cases.

84 pp. 8vo., price 1s. 6d., by post 1s. 8d.

WILLIAM F. CLAY, 18, TEVIOT PLACE, EDINBURGH.

